# Policy Perspectives of Dog-Mediated Rabies Control in Resource-Limited Countries: The Ethiopian Situation

**DOI:** 10.3389/fvets.2020.00551

**Published:** 2020-09-02

**Authors:** Tariku Jibat Beyene, Monique Mourits, Jeanette O'Quin, Samson Leta, Joaquin Baruch, Henk Hogeveen

**Affiliations:** ^1^Department of Veterinary Preventive Medicine, College of Veterinary Medicine, The Ohio State University, Columbus, OH, United States; ^2^Business Economics Group, Wageningen University, Wageningen, Netherlands; ^3^College of Veterinary Medicine and Agriculture, Addis Ababa University, Addis Ababa, Ethiopia; ^4^Department of Diagnostic Medicine and Pathobiology and Center for Outcomes Research and Epidemiology, College of Veterinary Medicine, Kansas State University, Manhattan, KS, United States

**Keywords:** economics, Ethiopia, rabies, public health, health policy

## Abstract

One Health disease-control programs are believed to be most effective when implemented within the population transmitting the disease. The World Health Organization (WHO) and partners have targeted the elimination of dog-mediated human rabies by 2030 primarily through mass dog vaccination. Mass vaccination, however, has been constrained by financial resource limitations. The current owner-charged dog vaccination strategy, used in most resource-limited countries like Ethiopia, has not reached the minimum coverage required to build population immunity. Dog vaccination is non-existing in most rural areas of Ethiopia, and coverage is <20% in urban areas. Although the health and economic benefits of rabies elimination outweigh the costs, the direct beneficiaries (public in general) and those who bear the costs (dog owners) are not necessarily the same. In this perspective paper, we aggregate evidence on the socioeconomic burden of rabies in Ethiopia as well as the implications for potential opportunities to control the disease and possibilities to obtain the required funding sources for evidence-based interventions in the control of rabies in Ethiopia.

## Introduction

Rabies is among the oldest infectious diseases known to man, and it carries the highest case fatality rate (1). Every year, about 60,000 people die due to rabies, equaling 3.7 million disability-adjusted life years (DALYs). The disease additionally causes an economic loss of around 9 billion USD globally (2). All mammals are susceptible to rabies, and domestic dogs remain the primary source of the disease to other dogs, humans, livestock, and wildlife (3). An estimated 99% of human cases globally are due to a bite from a rabid dog (1).

Countries in the Americas and Europe have eliminated the disease in domestic dogs through vaccination. In resource-limited countries of Africa and Asia, efforts to control rabies have progressed over the past several years and have accelerated following the global initiative to eliminate dog-mediated human rabies by 2030 set by World Health Organization and partners (4). This initiative has also motivated additional funding from international and charitable organizations to support the rabies control efforts of governments, especially in resource-limited settings. In most parts of Africa, however, minimal action has been undertaken (1).

The Stepwise Approach toward Elimination (SARE) is an assessment tool developed through a joint effort of the Food and Agriculture Organization (FAO) of the United Nations and the Global Alliance for Rabies Control (GARC) to provide a standard mechanism for countries to assess their current rabies control efforts and to measure progress in eliminating dog mediated human rabies. Ethiopia appears to be at an early stage (5). The SARE assessment identified several critical gaps including a lack of quantitative evidence on the burden and poor inter-sectoral collaboration between public health, animal health, and wildlife authorities. Following the first SARE assessment in 2016, the country established a One Health Working Group in the country with representatives from public health, animal health, and wildlife authorities, along with CDC, FAO, and Ohio State University; this included a Rabies Technical Working Group that developed a national rabies control and elimination strategy for the country (6). Although regions like Addis Ababa already mandate rabies vaccination requirements in place, in all administrative regions, canine vaccination is voluntarily-based and owner charged. Vaccination coverage varies from 18% in urban areas to almost non-existent in rural areas. These coverages are far lower than the 70% recommended minimum coverage to prevent rabies outbreaks (7). Although mass vaccination of dogs is a proven and cost-effective means of rabies control, there is a lack of motivation from owners and an inadequate intervention from local governments due to a lack of political will and resources (8).

Public and animal health authorities use disease burden metrics to set priorities in health investments (9). Often, these metrics do not consider all aspects of the socioeconomic burden of the disease. For instance, most of the burden studies focus on a human health perspective, and there is a paucity of data available on the health and economic impacts of rabies on livestock, wildlife, and animal welfare. Part of the problem is the lack of recorded and reported health data at human and veterinary health centers.

In this article, we summarize results from studies generated from registered rabies exposure and death cases of humans as well as from estimates using innovative data collection methods including contact tracing and participatory approaches to obtain the best possible estimate of the health and economic impact of rabies in humans and livestock in Ethiopia. Thus, the objective of this article is three-fold, (1) to summarize the burden of rabies in Ethiopia, (2) to indicate the potential benefit of vaccination control practices, and (3) to detail potential mechanisms to fund dog vaccination campaigns in resource-poor countries like Ethiopia.

## Health and Economic Burden of Rabies

Registered rabies cases, both by veterinary and human authorities, are underreported in many African and Asian countries. Consequently, officially registered data on rabies underestimates the true burden of the disease (10–13). In Ethiopia, no official mechanisms exist for public reporting of dog bites or rabies-related deaths unless people report while seeking medical treatment in health centers. In rural areas, the preferential use of traditional/spiritual healers might also contribute to the reduced level of post-exposure prophylaxis (PEP) (14). Based on registered health records, the annual human rabies exposure rate (based on refined exposure risk assessment performed following rabies suspected animal bite) per 100,000 population has been estimated to range from zero to 40 (15, 16). To account for underreporting, Beyene et al. (17) conducted an extensive survey-based case search, also known as contact tracing. The contact-tracing method has been demonstrated to give access to unregistered rabies exposures where exposure and or death cases are not fully registered (18, 19). Beyene et al. used registered cases obtained from health centers as a starting point to search for unregistered exposure cases in three representative districts. Results indicated that about 23% of the exposure cases (bitten by potentially rabid animal) did not seek medical attention. Accordingly, the annual suspected rabid dog exposures, which was based on the six criteria for rabies diagnosis in living dogs (20), were estimated to be 135, 101, and 86, resulting in 1, 4, and 3 deaths per 100,000 population within the studied urban, rural highland, and rural lowland districts, respectively. A treatment was assumed to be sufficient, adherence, only if the individual received the minimum recommended doses (at least 14 out of the 17 doses) of nervous tissue made PEP. Extrapolation of the district results to the national level using data from the country's national statistics on human population distribution in urban and rural districts as well as probabilities of disabilities and or deaths across ages indicated an annual estimate of ~3,000 human deaths resulting in about 194,000 DALYs per year as well as 97,000 exposed persons requiring on average 2 million USD treatment costs per year countrywide (1, 17). Twenty three percent of total human exposure cases included in the study were unreported and identified through the contact tracing. These findings suggest that relying on self-presentation for medical treatment will fail to reach ~1/4 of exposure cases. Communities should be encouraged to report dog bites, and active investigation of all known bites by appropriate authorities would be expected to identify additional exposures that require treatment, thereby saving lives. In 2001, WHO issued a resolution for the complete replacement of nerve tissue vaccines with cell-culture rabies vaccines. However, sheep brain-derived rabies vaccine is still being manufactured and used for most exposed patients in Ethiopia. This rabies vaccination has shown to cause disabilities and associated with costly indirect expenses as it requires up to 17 doses to complete full dose (17). Current initiatives of the Ethiopian government to invest in upgrading the facilities required to produce a safer and effective cell culture-based anti-rabies vaccine in line with WHO recommendation has to be encouraged (21).

Governments often use Disability-Adjusted Life Years (DALY) or Quality-Adjusted Life Years (QALY) estimations to rank diseases and to set priorities for health investments (22). Global funds also often prioritize public health-related grants following the DALY/QALY approach (23). Although rabies has a case fatality rate of nearly 100%, it is not on the top list of 25 most common diseases in countries like Ethiopia where diseases like malaria with a higher DALY/QALY burden prevails (24). However, the burden of zoonotic diseases such as rabies encompasses not only DALYs but also productivity/income losses, treatment-related costs, and societal costs in terms of psychological and emotional anxiety. The impacts are magnified in areas with poor access to PEP and in impoverished and remote rural communities. Rarely considered are the effects on livestock production threatened or endangered wildlife species (2, 23).

For the majority of Ethiopians, livestock is a direct source of livelihood, in terms of food and income. While crop output represented 32% of the country's GDP, about 80% of Ethiopian farmers use animal traction to plow their crop fields (24–26), and their crop production is affected when their oxen are diseased and lost due to rabies. Rabies outbreaks among the endangered Ethiopian wolf have nearly driven them to extinction (27). As such, the use of only DALY/QALY measure or the human health burden to set priorities in health investments is not serving the overall societal interest in the best way; a broader approach accounting for a more holistic assessment of the rabies burden is necessary.

## Economic Burden In The Ethiopian Livestock Sector

In Ethiopia, estimates on the burden of rabies in livestock are almost non-existent, except sporadic case reports (11, 12, 27). A recent attempt to evaluate the burden of rabies in cattle using a systematic approach was conducted in two systems of subsistence livestock farming systems, using a participatory approach. In this study, cattle rabies incidence rates at herd level were 21 and 11% for the mixed crop livestock and pastoral production systems, respectively. The incidence rate at cattle level was the same 2% in both systems. The annual national loss due to rabies in cattle alone was estimated to be 210 million USD per year (28). This is consistent with an economic model that predicted the financial loss to be between 10 and 412 million USD per year (2). The economic burden of rabies in cattle is not evenly distributed; it is especially severe for farmers in pastoral production systems who rely on cattle for much of their livelihoods (29).

## Burden on Wildlife Conservation, Research, and Tourism

Rabies threatens many of the endangered species of wildlife. The Ethiopian wolf is one of these species whose number is decreasing at an alarming rate due to rabies and other viral diseases (30). Although scarce literature documented the contribution of wildlife to the Ethiopian economy, wildlife-based tourism contributes significantly to the economy of Kenya, Tanzania, and Uganda (31–33). As populations decline to make them more difficult to locate, research, and wildlife-centered tourism could decrease. Additionally, tourism in general may be reduced due to fears of contacting a rabid dog.

## Implications on The Economics of Control

Nearly all cases of rabies in Ethiopia originate from dogs. Many countries have demonstrated that canine mass vaccination will reduce the burden of rabies in humans as well as in livestock and wildlife (2, 30, 34). Reducing disease also improves animal welfare. The cost-effectiveness for dog vaccination has been demonstrated in various countries of Africa and Asia (29, 31). Specific parameters like dog population and livestock density affect cost-effective vaccination coverage. A global needs assessment study estimated dog population in Ethiopia to be 11.7 million using extrapolation of dog per human population data (35).

In Ethiopia, Beyene et al. (8) estimated the cost-effectiveness of mass vaccination in representative urban and rural districts while accounting for human health impacts as well as livestock impacts. This particular study simulated over the period of 5 years identified vaccination coverages of 70 and 80% to be the most likely to provide the greatest net health benefits in urban and rural districts, respectively. The exclusion of cattle related losses in the cost-effectiveness analysis, for the rural district scenario, shifted the cost-effective coverage from 80 to 50%, suggesting that the economic burden of rabies in cattle represents a relevant financial incentive for canine vaccination. Based on a more inclusive notion of disease burden, the cost-effectiveness analysis for the rural district showed that all tested vaccination scenarios varying from 10 to 90% coverage resulted in a positive net monetary benefit. In other words, the cost of the mass vaccination campaign is less than the total financial loss associated with rabies, which includes cattle-related rabies. On the other hand, the active investigation to identify other bite victims also comes at an reasonable additional cost to the program, which was not included in the cost accounting of the study (8). Similar studies need to consider at least costs of risk-based investigation, although implementation has been difficult for many countries including Ethiopia where funding for dog vaccination is limited.

In this study, elimination would not be achieved within the first 5 years but the level of coverages would protect an outbreak and sporadic rabies could occur. Consistent and higher coverage would be required to eliminate rabies virus transmission and low coverages would not eliminate the disease in the dog population that requires sustained vaccination costs indefinitely. The net benefit could be even higher if tourism losses secondary to rabies fears as well as conservation of wildlife could be included in the analysis.

## Who Should Pay for Dog Vaccination?

For an annual cost-effective canine mass vaccination campaign with a coverage of 70% in urban and 80% in rural, the total investment for Ethiopia is estimated to be 17.5 million USD/year, in the order of 0.2$/dog per year (8). An investment of 17.5 million USD is a big investment for the Ethiopian government to allocate to rabies control compared to the amount of the budget allocated to the health sector in general. A comparable estimate has been reported by the team of researchers from CDC, WHO, and FAO on needs assessment and Alternatives for Progress Based on Dog Vaccination to meet the 2030 target of dog-mediated human rabies elimination for Ethiopia to be $135 million for a period of 2017 to 2030 (35). The Ethiopian government allocated about 7–11% of the total fiscal budget to the health sector, which equals 388 million USD (36). Though the Ethiopian government determined that rabies was a top-priority zoonotic disease in 2015 (37), sufficient funding to conduct an 17.5 million mass vaccination campaign have not been allocated to accomplish this goal. On the other hand, the budget estimate assumes that every community in Ethiopia requires dog vaccination. This might be an overestimate as there could be communities that would not require vaccination due to the very low risk of transmission perspective pertaining to the lower population density of dogs as demonstrated by a study conducted in Uganda (38). Similar studies that could identify areas with their potential risk of rabies would be helpful for budget allocation purposes. Utilizing external resources like international partners, including CDC and Ohio State University, which provide training for staff to plan and conduct mass vaccination campaigns and cover part of vaccination cost, could help; however, consistent funding is needed for desirable outcome (39).

Even though the benefits of rabies elimination outweigh the costs of control, the beneficiaries (general public and livestock owners) and those who bear the costs (dog owners) are not necessarily the same. The benefits in terms of improved public health, reduced costs of post-exposure treatments, and better cattle health are not distributed to the public equally. Given the current situation in Ethiopia, insisting on owner-charged dog vaccination is expected to result in far lower coverage. This is supported by a review article of the literature on mass vaccination in Africa which found that none of the fee required projects reached the 70% target vaccination coverage, while the free campaigns consistently achieved higher vaccination rates (40). The challenges in urban districts are exacerbated by the presence of free-roaming dogs (owned and/or without owners) compared to rural areas, which are less likely to be vaccinated in owner-fee campaigns (41). Effective rabies vaccination of dogs would require government involvement in covering the associated costs. A partial dog–owner contribution could also be applied as demonstrated in Asia (42).

Governments could follow financing strategies such as joint financing including the “separable costs–remaining benefits” method of cost-sharing (43) to allocate the expenditures to both sectors proportional to the benefits gained by both sectors, for instance, veterinary, and public health sectors. Such a proportional allocation of resources was also simulated for Rift Valley Fever control in Kenya and Brucellosis control in Mongolia (37, 38). A more sustainable rabies control program was demonstrated in Bohol (Indonesia) through legalizing the control framework (i.e., compulsory dog registration to establish responsible pet ownership and accountability in combination with mass vaccination to establish dog herd immunity), mobilizing local resources and involving the local community (44). Alternatively, synergistic funding options for vaccination campaigns could include a loan through development-impact funding, where investments are paid back over several years once savings are noticed as a result of benefits from disease control (45). Potential savings result from a reduced need for post-exposure prophylaxis and wound treatments and other related healthcare facility resource expenses. This approach is a form of social impact bond, whereby initial costs of disease control are supported by private investors and repaid by donors and governments once agreed outcomes are achieved. These funding mechanisms were demonstrated to work for the control of sleeping sickness in Uganda (46). Given that rabies has a readily available vaccine that is highly effective, and it requires a relatively large public investment, rabies control would be a perfect candidate for such financing in countries like Ethiopia. Effective control of rabies would likely reduce human incidence leading to a significant reduction in PEP, which is currently an expenditure to the government (47). Although there would not be a direct monetary saving for the government as a result of the saving from reduced burden of the diseases in livestock, it provides an indirect societal benefit in times of food insecurity. Short-term saving for the government would be from reduced expenditures related to PEP production and or import could be used to pay back the bonds.

To better prepare the country to conduct mass vaccination of dogs, various partners including Global Health initiative at Ohio State University and CDC have been building capacity and, through multiple training efforts, have increased vaccination coverages in some localities. Collaboration between public and animal health authorities in terms of sharing expertise and resources should be developed. Establishment of such units at different administrative levels, including practicing veterinarians and medical doctors, would improve communication about specific risks and could contribute to practical One-Health-oriented cooperation. Such collaboration between human and animal healthcare professionals can also avoid unnecessary public expenditure due to post-exposure treatment in the case that biting dogs are investigated and found not to be rabid. Active investigation of all dog bites can lead to the identification and treatment of other persons who were exposed as well as verify the rabies status of the animal. Operationalizing such a cross-sectoral agenda could be challenging in most countries (48). However, some countries, Kenya and Haiti, for instance, have successfully established a zoonotic disease unit and implemented (48, 49). Apart from rabies, in Ethiopia, some of the top-listed diseases in terms of health burden like diarrheal diseases are partly zoonotic (22), indicating a broader benefit for operationalizing a One Health approach.

## Conclusion

In this perspective article, we demonstrated that through uncovering evidence on the multifaceted burdens of rabies using unconventional methods; it is possible to generate evidence that contributes support toward a cross-sectoral political and financial approach to canine mass vaccination. Particularly, in rural livestock-owning communities, the impact of rabies on cattle health and productivity, in addition to its public health impacts, could be viewed as an additional incentive for governmental support of canine vaccination efforts. Rabies has already been declared by the Ethiopian government to be a priority zoonotic disease. Considering a broader definition of the evaluation of disease burden could also help justify the funds needed for rabies effective control. Most of these are also consistent with findings from global and regional rabies burden estimation models.

Despite availability of Ethiopian and global evidence on rabies burden and cost-effective options, little improvements have been made on practical interventions of rabies by the Ethiopian government over the past years. While the authors recognize the financial challenge to implement intervention, the country needs to further explore a way to operationalize the principles of One Health involving various sectors. In addition, we strongly believe that (1) it is not reasonable for dog owners to shoulder the majority of the cost for rabies vaccination efforts aimed to protect the entire population, (2) when primary vaccination efforts rely on dog owners to pay for rabies vaccination, even if they could all afford it, vaccination coverage rates high enough to interrupt dog-to-dog transmission of rabies will not be achieved; it is recommended that mass rabies vaccination of canines be conducted through free or partial cost to owner programs that target both owned and free-roaming dogs. Furthermore, One Health collaboration in other areas including dog bite investigation and public awareness should be considered to control rabies.

## Data Availability Statement

The original contributions presented in the study are included in the article/supplementary material, further inquiries can be directed to the corresponding author/s.

## Author Contributions

TB, MM, and HH conceived the idea and organized the literature review. TB analyzed the data and interpreted the results. TB, MM, HH, JO'Q, SL, and JB wrote the draft of the manuscript and completed the final version for submission. All authors approved the final version of the manuscript before submission.

## Conflict of Interest

The authors declare that the research was conducted in the absence of any commercial or financial relationships that could be construed as a potential conflict of interest.
